# Expression of Autophagy-Related Factors LC3A and Beclin 1 and Apoptosis-Related Factors Bcl-2 and BAX in Osteoblasts Treated With Sodium Fluoride

**DOI:** 10.3389/fphys.2021.603848

**Published:** 2021-07-01

**Authors:** Lin Xu, Chaonan Deng, Ying Zhang, Lina Zhao, Yan Linghu, Yanni Yu

**Affiliations:** ^1^Guiyang Maternal and Child Health Care Hospital of Guiyang, Guiyang, China; ^2^Department of Pathology, The Affiliated Hospital of Guizhou Medical University, Guiyang, China; ^3^Department of Pathology, Guizhou Medical University, Guiyang, China

**Keywords:** autophagy, apoptosis, osteoblasts, light chain 3 alpha, Beclin 1, B-cell lymphoma 2, BCL2 associated X, 3-methyladenine

## Abstract

**Objective:**

This study aims to analyze the expressions of autophagy-related factors light chain 3 alpha (LC3A) and Beclin 1 and apoptosis-related factors B-cell lymphoma 2 (Bcl-2) and Bcl-2-associated X (BAX) in primary osteoblasts treated with sodium fluoride (NaF).

**Methods:**

Osteoblasts were extracted from Sprague-Dawley rats and treated with 0, 2.5, 5, and 10 mg/L NaF solutions, followed by 10 mmol/L 3-methyladenine (3-MA) for 24 h. The apoptotic rate was determined by flow cytometry, and the expressions of the autophagy- and apoptosis-related factors were measured by western blotting and real-time quantitative polymerase chain reaction.

**Results:**

The mRNA expressions of LC3A, Beclin 1, and BAX in the NaF-treated osteoblast group were higher than those in the control group, while the protein expressions of these factors in the NaF-treated group were significantly higher than those in the control group. However, the Bcl-2 protein expression in the NaF-treated osteoblasts was significantly decreased compared to that in the control cells. After the 3-MA treatment, the protein expressions of LC3A, Beclin 1, and Bcl-2 were significantly decreased compared with those of the NaF-treated group, whereas the expression of BAX increased. Moreover, the apoptosis rate was increased after the addition of the 3-MA inhibitor.

**Conclusion:**

NaF stimulation promoted autophagy and apoptosis of the osteoblasts, suggesting the involvement of fluoride damage in these processes.

## Introduction

Endemic fluorosis is a global disease (Wei et al., 2019). While physiological doses of fluoride are beneficial to the human body and can promote bone growth and development, excessive fluoride intake leads to its accumulation in a variety of organs, eventually causing fluorosis (Wang et al., 2019a). The main symptoms of the disease are only fragments of the damage, however. As the study of skeletal fluorosis has been thorough, the deleterious effects of fluoride on bone are well known (Wang et al., 2019b, 2020). The main performance for osteoblast numbers increases mast cell body, which is the basis for extensive cell damage and apoptosis (Ravanan et al., 2017).

Autophagy is a highly valued protective mechanism in the process of evolution (Antonioli et al., 2017; Ommati et al., 2019, 2020a,b). However, while it can remove damaged proteins and organelles to maintain the stability of the intracellular environment, in excess it can cause damage to organelles, an unstable internal environment, and eventually death (Cicchini et al., 2015). This may be one of the reasons for the pathological changes of osteomalacia, osteosclerosis, and osteoporosis in fluorosis. Light chain 3 alpha (LC3A) and Beclin 1 have been used as markers to detect autophagy activity (Ding et al., 2016). Apoptosis is regulated by many related genes, such as B-cell lymphoma 2 (Bcl-2) and Bcl-2-associated X (BAX) (Anesan et al., 2018).

The present study analyzes the key factors related to autophagy (LC3A and Beclin 1) and apoptosis (Bcl-2 and BAX) in the bone tissues of rats with chronic fluorosis. Osteoblasts extracted from the skulls of Sprague-Dawley rats were treated with autophagy-specific inhibitor 3-methyladenine (3-MA), and the expressions of the autophagy- and apoptosis-related factors in cultured osteoblasts were analyzed. The results indicate that autophagy and apoptosis may be jointly involved in the process of fluoride-induced bone-tissue damage.

## Materials and Methods

### Extraction, Identification, and Treatment of Osteoblasts

Sprague-Dawley rats were euthanized within 1 week of birth, and their skulls were removed and sterilized. The skull was cut into 0.5 cm^3^ pieces in a petri dish filled with low-sugar DMEM. Then 1 mL of 0.25% trypsin was added to tissues for 20 min before adding serum to stop trypsin digestion. The supernatant was removed and added with 10 mL of 1.0 g/L type I collagenase for 90 min. Then the samples were centrifuged at 1,000 r/min for 10 min, washed with PBS, and added low-sugar DMEM containing 10% fetal bovine serum and 1% penicillin-streptomycin. Cells were then inoculated into a 25 cm^2^ culture flask containing Dulbecco’s modified Eagle’s medium with 10% fetal bovine serum and 1% penicillin/streptomycin solution. Cells were then placed in an incubator, maintained at 37°C with 5% CO_2_ and 95% saturated air, before being subcultured at 1:3. The alkaline phosphatase staining kit (Abcam, Cambridge, MA, United States) was used to identify the osteoblasts (Ehnert et al., 2015). Cells were then fixed in the fixation solution (provided in the kit) according to the manufacturer’s instructions for 30 s. Then the samples were sliced, incubated in the dark for 30 min, stained with Mayer’s hematoxylin solution for 10 min, dried, and sealed with neutral gum.

Under a microscope, the density of the primary osteoblasts reached 80%. Pre-configured 0, 2.5, 5, and 10 mg/L sodium fluoride (NaF) solutions were added to the culture bottles and incubated for 24, 48, and 72 h. Following this, the cells were transplanted into 96-well plates; 10 μL of Cell Counting Kit-8 (CCK-8) solution was added to each well and read on a Rayto RT-6000 microplate reader (Rayto Life and Analytical Sciences Co., Ltd., Shenzhen, China) at 570 nm. To determine a suitable concentration and time period for fluoride dyeing, different concentrations of 3-MA (1.25, 2.5, 5, and 10 mmol/L) were added. After 12, 18, and 24 h of equal treatment, a CCK-8 assay was performed to screen the time and concentration of the studied autophagy inhibitors.

The experimental protocol was approved by the Animal Care Welfare Committee of Guangzhou Medical University (1603070) and performed following the *Guide for the Use of Laboratory Animals*.

### Determination of Apoptosis Rate

After cells were collected in an Eppendorf tube, 1.5 density was added to 1 × 10^6^/mL and centrifuged at 1,000 RPM/min for 5 min. Then cells were resuspended with 1 mL pre-cooled PBS, transferred to a new Eppendorf tube, and centrifuged again at 1,000 RPM/min for 5 min. The clear liquid was carefully removed, and 100 μL (including one heavy osteoblast cell suspension) was added to the cells, which were then transferred to a 5 mL falcon tube, into which 10 μL (including one Annexin V-FITC blending) was added. The material was placed on ice in the dark for 15 min, after which 5 PI of dye was added. The material was then incubated for a further 5 min on ice in the dark, and 400 μL binding buffer was added to re-suspend the osteoblasts for flow cytometry analysis (Han et al., 2018).

### Real-Time Quantitative Polymerase Chain Reaction (qPCR) Analysis

Real-time qPCR was used to detect the messenger RNA (mRNA) expressions of LC3A, Beclin 1, Bcl-2, and BAX in the cells in the control group, fluoride-stained group, and autophagy inhibitor. The total RNA in each group was extracted using TRIzol^®^ reagent according to the manufacturer’s instructions. The RNA concentration was read on a protein/nucleic acid quantitative analyzer to test its purity. The A260/280 ratios of the pure RNA samples were 1.8–2.0 RNA. The RNA samples were loaded onto 1.5% agarose gel electrophoresis and observed using a gel imaging system. The synthesis of the first strand of complementary DNA (cDNA) was then completed according to the manufacturer’s instructions (RDRT-100RXN, Sigma-Aldrich, St. Louis, MO, United States). In brief, 5 μm of the RNA samples and 181 μm of oligo (dT) primer (a total of 12 μm of high-purity water) without nuclease were taken, incubated at 65°C for 5 min, and cooled on ice. After being centrifuged, the primer sequences of LC3A, Beclin 1, Bcl-2, and BAX were designed, after which 4 μM of 5 × reaction buffer, 20 μM of RiboLock^TM^ RNA enzyme inhibitor, and 10 mM of deoxyribonucleoside triphosphates were added. A total of 2 μm of RevertAid^TM^ and 200 μm/mul of Moloney murine leukemia virus reverse transcriptase were mixed and incubated at 42°C for 60 min before being heated at 70°C for 5 min and preserved at –80°C.

The primer sequences of LC3A, Beclin 1, Bcl-2, and BAX were designed as shown in Supplementary Table 1. A total of 10 μL of SsoFast^TM^ EvaGreen Supermix (Bole instrument with fluorescent reagent), 0.3 μL of upstream primer, 0.3 μL of reverse primer, 8.4 μL of distilled deionized water, and 1 μL cDNA 1 were added to the system. The quantitative analysis was performed using a fluorescence-based quantitative PCR instrument via pre-denaturation at 95°C for 30 s and then 10 s, followed by annealing at 60 and 72°C for a further 30 s. The standard procedure for collecting the fluorescence was 40 cycles. After pre-denaturation at 95°C for another 15 s, the process of fluorescence melting curve collection was carried out: between 60 and 95°C, the fluorescence signal was collected every time the temperature rose by 0.5°C, and the dissolution curve was analyzed.

### Western Blotting

The study used western blotting to detect the expression of LC3A, Beclin 1, Bcl-2, BAX, and protein with cracking liquid (pyrolysis liquid/PMSF ratio = 100:1). The extraction of the osteoblast protein groups was undertaken according to the instructions of a standard-curve bicinchoninic acid protein-detection kit. The standard enzyme instrument was measured using optic density value. The sample protein concentrations were calculated and separated according to molecular weight. The manufacturing rubber was extracted and loaded onto the electrophoresis separation gel and then transferred to an FVDF membrane with 5% skimmed milk powder. The PVDV membrane was stained with the following primary antibodies: LC3A (cat no. 4599S, Cell Signaling Technology, Danvers, MA, United States; 1:2,000 dilution), Beclin (cat no. ab62557, Abcam; 1 1:1,500 dilution), Bcl-2 (cat no. ab18210, Abcam; 1:1,000 dilution), and BAX (cat no. ab32503, Abcam; 1:1,500 dilution). Horseradish peroxidase-labeled secondary antibody (1:2,000) was then added and developed.

### Statistical Analysis

Data were expressed as mean ± standard deviation from three independent experiments, each performed in triplicate. SPSS 22.0 software was used for statistical analysis. One-way analysis of variance (ANOVA) was performed to compare the variable means among multiple groups, and Student’s SNK was used in *post hoc* tests to compare means between groups following a significant ANOVA.

## Results

### Identification of Fluorinated Osteoblasts in a Culture

**FIGURE 1:**
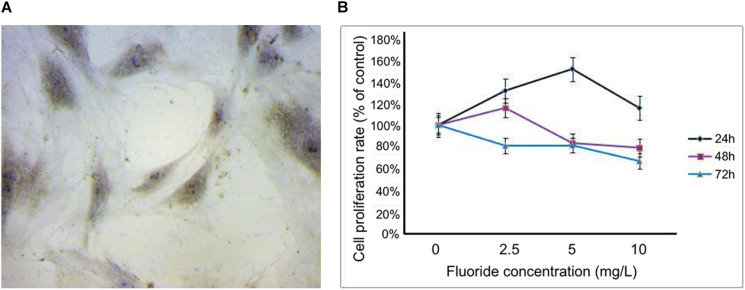
Primary rat osteoblasts and cell proliferation rate after treatment with NaF. **(A)** Dark-brown granules in the cytoplasm of osteoblasts were observed after alkaline phosphatase staining. **(B)** Cell proliferation rate was measured using a cholecystokinin octapeptide assay at 24, 48, and 72 h after treatment, with fluoride at different concentrations (0, 2.5, 5, and 10 mg/L).

When the primary cells were transferred to the fourth generation, the osteoblasts adhered to the wall with a good spindle shape, round nucleus, and good refractive properties. Upon alkaline phosphatase staining, dark-brown granules were observed in the cytoplasm (Figure 1A). The cell survival rates at different concentrations (2.5, 5, and 10 mg/L) and time periods (24, 48, and 72 h) were obtained using the CCK-8 assay. At concentrations of 2.5, 5, and 10 mg/L, the cell survival rate at 24 h were 131.38, 151.82, and 115.80%, respectively. At 48 h, they were 115.87, 83.31, and 79.06%, respectively, and at 72 h they were 80.91, 81.22, and 66.66%, respectively. Based on these results, 24 h was selected as the best time period, and 2.5 and 5 mg/L, respectively, were selected as the low-fluoride and high-fluoride concentrations. Based on an inhibition rate of 50%, the optimal concentration of 3-MA was selected as 10 mmol/L and the treatment time as 24 h (Figure 1B).

**FIGURE 2:**
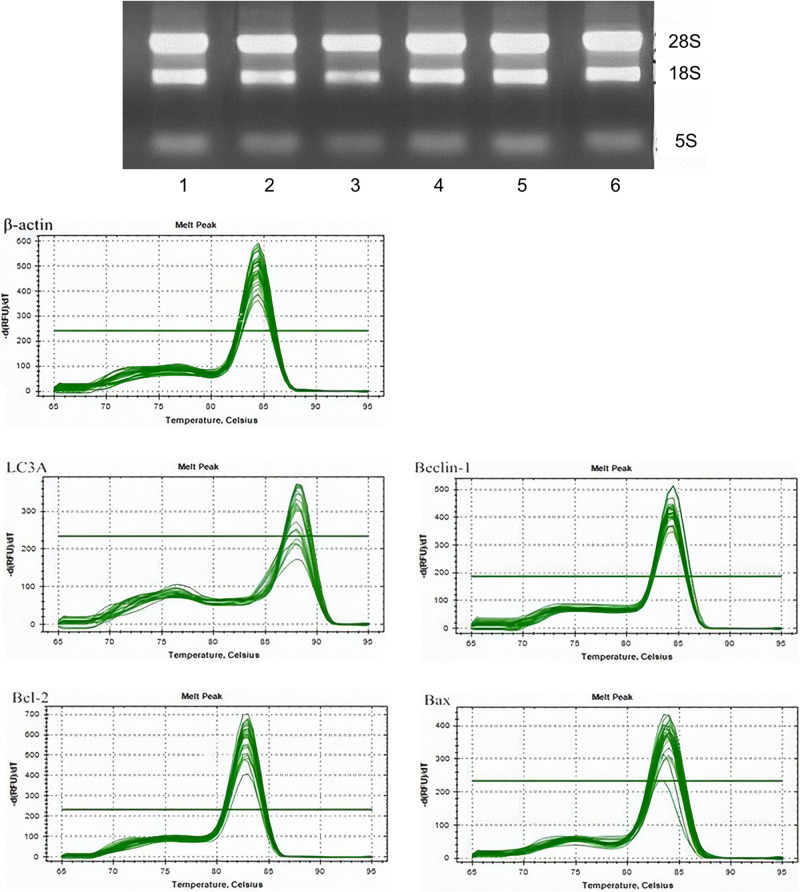
The messenger RNA (mRNA) expressions of LC3A, Beclin 1, Bcl-2, and BAX in osteoblasts. The mRNA expressions of LC3A, Beclin 1, Bcl-2, and BAX in the following groups of osteoblasts were assessed by real-time quantitative polymerase chain reaction: control group; low-fluoride (2.5 mg/L) group; high-fluoride (5 mg/L) group; control + 3-MA (10 mmol/L) group; low-fluoride (2.5 mg/L) + 3-MA (10 mmol/L) group; high-fluoride (5 mg/L) + 3-MA (10 mmol/L) group. * indicates *P* < 0.05 compared with the control group; # indicates *P* < 0.05 compared with the low fluoride group; & indicates *P* < 0.05 compared with the high fluoride group.

The RNA expression was then measured. The experimental results suggest that there were no significant differences between the mRNA content in the control and 3-MA groups (*P* > 0.05). Dyeing LC3A in the fluoride group showed that the Beclin 1 mRNA content increased with the concentration of fluoride dye. The mRNA content in the fluoride-stained group was lower than that in the fluoride-stained group at the same concentration, and the difference was statistically significant (*P* < 0.05). The expression of the Bcl-2 mRNA in the fluoride-stained group decreased, and it was significantly lower in the low-fluoride group than in the high-fluoride group. It was higher in the BAX group than in the control group, with the most obvious increase being in the low-fluoride group (Figure 2).

**FIGURE 3:**
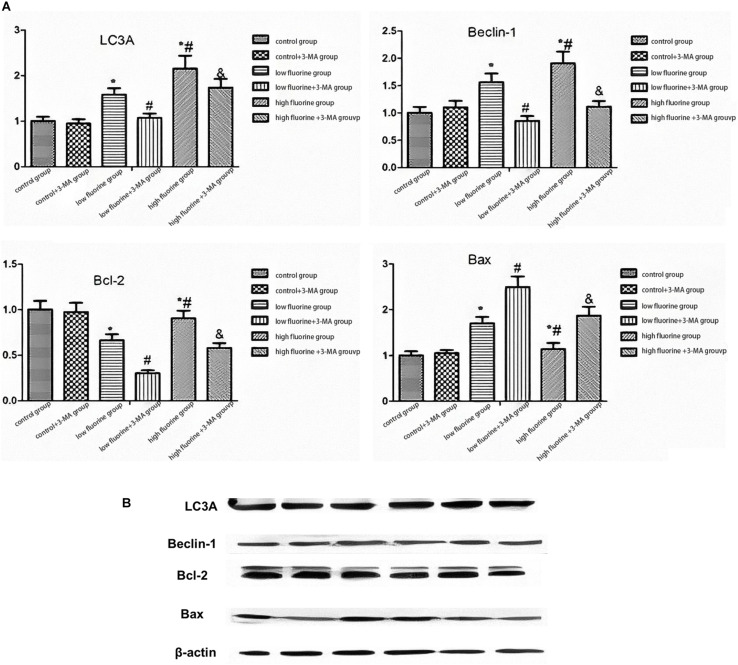
The protein expressions of LC3A, Beclin 1, Bcl-2, and BAX in osteoblasts. The protein expressions of LC3A, Beclin 1, Bcl-2, and BAX in the different groups of osteoblasts were assessed by western blot. **(A)** Representative blots and **(B)** quantitative analysis are shown. From left to right: control group; low-fluoride (2.5 mg/L) group; high-fluoride (5 mg/L) group; control + 3-MA (10 mmol/L) group; low-fluoride (2.5 mg/L) + 3-MA (10 mmol/L) group; high-fluoride (5 mg/L) + 3-MA (10 mmol/L) group. * indicates *P* < 0.05 compared with the control group; # indicates *P* < 0.05 compared with the low fluoride group; & indicates *P* < 0.05 compared with the high fluoride group.

Figure 3 shows the protein expression of LC3A, Beclin 1, Bcl-2, and BAX. As the fluoride dye concentration increased, the protein expression of Beclin 1 was gradually increased. The BAX protein expression also increased, but the increase was most pronounced in the low-fluoride group. As the fluoride dye concentration of the protein expression in the low-fluoride group increased, the drops in Bcl-2 expression showed the most obvious decrease. Compared with the protein expressions of LC3A, Beclin 1, and Bcl-2 in the fluoride-stained group, those in the fluoride-stained group and 3-MA group decreased, while the protein expression of BAX increased. The differences between the groups were statistically significant (*P* < 0.05).

### Apoptosis of Fluorinated Osteoblasts

**FIGURE 4:**
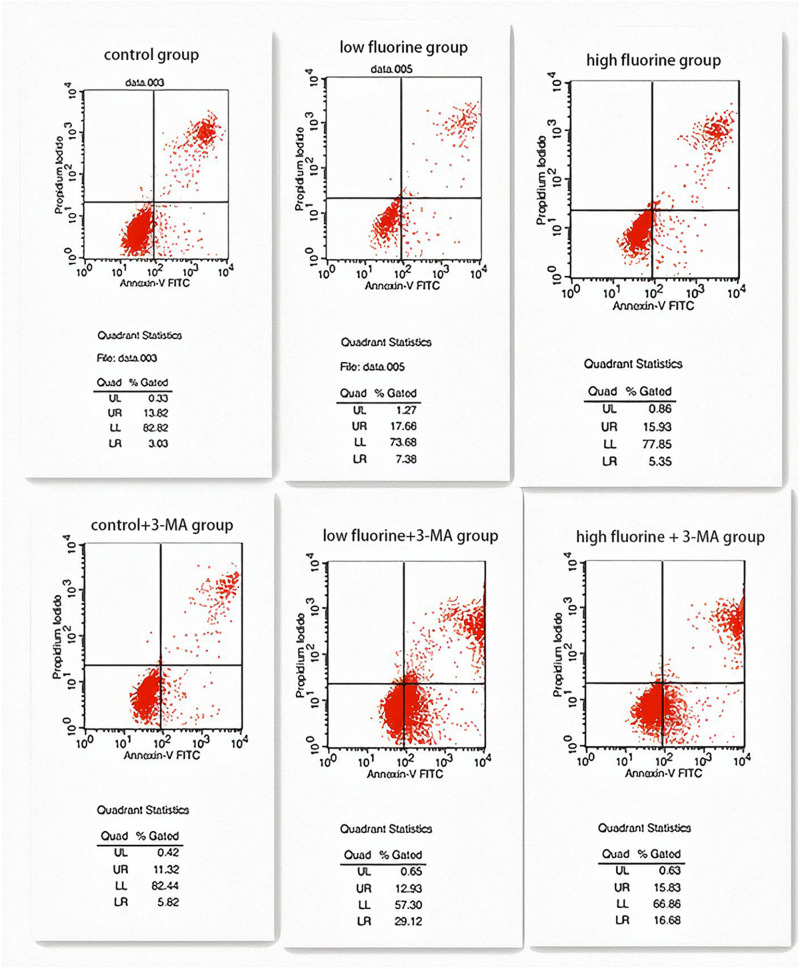
Apoptosis of cultured osteoblasts. The apoptosis rates of the following groups of osteoblasts were detected by flow cytometry: control group; low-fluoride (2.5 mg/L) group; high-fluoride (5 mg/L) group; control + 3-MA (10 mmol/L) group; low-fluoride (2.5 mg/L) + 3-MA (10 mmol/L) group; high-fluoride (5 mg/L) + 3-MA (10 mmol/L) group.

The apoptosis rates of the control group and the control 3-MA group were very similar (16.85 and 17.14%, respectively), and there was no statistical significance between them (*P* > 0.05). The apoptosis rate of the low-fluoride group (25.04%) was higher than that of both the control group and the high-fluoride group (21.28%), and the differences between the groups were statistically significant (*P* < 0.05). The apoptosis rate increased after the 3-MA inhibitor was added and was most obvious in the low-fluoride 3-MA group (42.05%) and high-fluoride 3-MA group (32.51%) (*P* < 0.05) (Figure 4).

## Discussion

Bone tissue is primarily composed of five types of cells: bone progenitor cells, osteoblasts, bone cells, bone-covering cells, and osteoclasts (Florencio-Silva et al., 2015). Osteoblasts are mainly active on the surface of bone tissue and have a more pronounced secretion function (Blair et al., 2017). They synthesize and secrete bone-glue fibers and the organic matrix. They are important in the process of bone formation due to their synthesis, secretion, and mineralization functions (Zhao, 2012). Proliferation, extracellular matrix maturation, extracellular matrix mineralization, and apoptosis are the four stages of bone formation. The primary function of osteoblasts is to complete osteogenesis (Rutkovskiy et al., 2016). Osteoblast proliferation is mainly responsible for the replication and average distribution of the cytoplasm and nucleus, thereby increasing the number of cells (Wang et al., 2011). Osteoblasts have been studied *in vitro* for over 40 years (Cheng et al., 2013). In 1964, (Peck et al., 1964) researched the use of collagenase digestion, successfully separating rat osteoblasts for the first time, and in 1975, Wong and Cohn (1975) developed a collagenase digestion method using multiple purifications. In the 1980s, Robey (Robey and Termine, 1985) used low-calcium cultivation of bone for osteoblasts.

It is well known that fluoride has a damaging effect on bones. Bones are the target organ of local fluorosis, and hyperplasia, osteomalacia, osteoporosis, ossification of periosseous soft tissue, and joint degeneration are the main manifestations of such problems (Chen et al., 2016). Previous studies have shown that active osteoblast function and accelerated bone transformation play important roles in the pathogenesis of skeletal fluorosis (Sun and Gao, 2008; Ma et al., 2013; Gu et al., 2014; Zhang et al., 2014). The active function of osteoblasts is an early and dominant link between them, mainly manifesting as cell-body hypertrophy and an increase in the number of osteoblasts (Li et al., 2014). Apoptosis is the basis of the extensive cell damage when endemic fluorosis occurs; the osteoblast damage caused by fluorosis is related to the cell apoptosis caused by fluoride ions, and the apoptosis mechanism is closely related to the permeability of the mitochondrial membrane and intracellular reactive oxygen species (Huang et al., 2012).

In a previous study, Yang et al. (2011) found that FIP200 protein could be removed from rat osteoblasts to inhibit autophagy, after which osteogenesis decreased, manifesting as poor bone growth and decreased bone mass. In the *in vitro* culture of osteoblasts, it was found that osteoblast mineralization was accompanied by increases in LC3A, indicating that autophagy was primarily involved in the mineralization and differentiation of osteoblasts. Thus, autophagy may be one of the main causes of bone-tissue lesions.

Sodium fluoride bidirectionally regulates the proliferation and differentiation of osteoblasts (Chen et al., 2019). A small dose promotes cell growth, while a large dose inhibits its growth, showing a dose-time effect, which may explain why fluorosis induces both osteomalacia and osteosclerosis. In the present study, osteoblasts from suckling rats were extracted and cultured *in vitro* with 2.5 and 5 mg/L NaF for 24 h to study cell proliferation. The proliferation in the fluoride-stained group was significantly increased compared with that in the control group, suggesting that low doses of fluoride could promote osteoblasts; these findings were consistent with those of other studies (Li et al., 2003; Meng et al., 2013). The question of how autophagy changes in osteoblasts under the action of a low dose of fluoride was then raised, and the study tested the changes in autophagy-related genes LC3A and Beclin 1 to explore the relationship between fluorosis and autophagy. It was found that fluoride could induce autophagy in osteoblasts; as the fluoride concentration increased, the level of intracellular autophagy gradually increased in a dose-dependent manner, further confirming one of the mechanisms of abnormal fluoride levels in osteoblasts upon bone injury in rats. The expression level of Bcl-2 in the osteoblasts decreased significantly in the low-fluoride group, while the expression level of BAX increased, suggesting that fluoride promotes the apoptosis of osteoblasts. Previous studies have reported that the osteoblast damage caused by fluorosis is related to the apoptosis reaction caused by fluoride ions (Deng et al., 2015). Yang et al. (2011) found that NaF could increase the permeability of the mitochondrial membrane of osteoblasts, downregulate the expression of Bcl-2 mRNA, upregulate the expression of BAX mRNA, and initiate the signaling pathway of mitochondrial apoptosis, which is consistent with the results of the present study. Overall, the study found that low concentrations of NaF promoted the proliferation of osteoblasts and the occurrence of autophagy and apoptosis.

The flow cytometry results of the present study indicated that osteoblast apoptosis did not increase, with the dyed fluoride concentration fluoride group’s apoptosis rate being higher than that of the control group – over 2.5 mg/L in the NaF-treated group compared with 5 mg/L in the NaF group. The BAX and Bcl-2 protein and mRNA expression in the fluoride dye group could have been due to the fact that a low concentration of fluoride promotes cell proliferation and apoptosis but not osteogenesis of the osteoblast proliferation cell apoptosis. The protein and mRNA expressions of autophagy-related genes LC3A and Beclin 1 in the fluoride-stained group increased with fluoride concentration. Compared with the protein and mRNA results of BAX and Bcl-2, it was found that the apoptosis expression level decreased when the autophagy expression level increased under the same conditions.

To further explore the relationship between autophagy and apoptosis, the present study adopted a specific inhibitor of autophagy, 3-MA, which mainly inhibits the activity of the PI3K protein in the autophagy channel, thus impeding the formation of autophagosomes and, thereby, the autophagy level. 3-MA is widely used in research on the inhibition of autophagy levels. The study found that the addition of 3-MA to the two groups had no significant effect on the growth or morphological changes of the osteoblasts nor on the protein or mRNA levels. The inhibitor was then added to low- and high-concentration fluoride-stained cells. It was found that the LC3A and Beclin 1 protein and mRNA expressions in the fluoride-stained 3-MA group decreased in comparison with those of the fluoride-stained group with the same concentration, indicating that 3-MA could inhibit the occurrence of fluoride-induced autophagy in osteocytes. Meanwhile, the Bcl-2 protein and mRNA expressions decreased, while those of BAX increased. It was observed that after the addition of 3-MA, autophagy was inhibited and apoptosis promoted. These results indicate that autophagy inhibits apoptosis following the administration of a low concentration of fluoride, suggesting that autophagy has a protective effect on osteoblasts.

## Conclusion

NaF stimulation was found to promote the autophagy and apoptosis of osteoblasts, suggesting that it is involved in the process of pathological osteoblast damage related to these processes.

## Data Availability Statement

The original contributions presented in the study are included in the article/Supplementary Material, further inquiries can be directed to the corresponding author.

## Ethics Statement

The animal study was reviewed and approved by Animal Experimental Ethical Inspection Form of Guizhou Medical University.

## Author Contributions

LX and YY conceived the idea, conceptualized the study, and drafted the manuscript. CD collected the data. YZ and LZ analyzed the data. YL reviewed the manuscript. All authors read and approved the final draft.

## Conflict of Interest

The authors declare that the research was conducted in the absence of any commercial or financial relationships that could be construed as a potential conflict of interest.
