# Supplementary material for: Expression of Autophagy-Related Factors LC3A and Beclin 1 and Apoptosis-Related Factors Bcl-2 and BAX in Osteoblasts Treated With Sodium Fluoride
Source: Front Physiol. 2021 Jul 1;12:603848. doi: 10.3389/fphys.2021.603848 (PMC8281676; doi:10.3389/fphys.2021.603848)
Supplement: Supplementary file 1 [file Table_1.docx]

Supplementary table 1 **The sequences of real-time PCR Primers**

| Gene name | primer sequence | Amplified fragment size |
| --- | --- | --- |
| LC3A | F:TTCGCCGACCGCTGTAA | 286bp |
|  | R:ATCCGTCTTCATCCTTCTCCT |  |
| Beclin-1 | F:AGTGGCGGCTCCTATTC | 108bp |
|  | R:GGACACCCAAGCAAGAC |  |
| Bcl-2 | F:CACGGTGGTGGAGGAAC | 254bp |
|  | R:ACAGCCAGGAGAAATCAAA |  |
| Bax | F:CAAACTGGTGCTCAAGGC | 146bp |
|  | R:TCCCGAAGTAGGAAAGG |  |
| Β-actin | F:CACGATGGAGGGGCCGGACTCATC | 252bp |
|  | R:TAAAGACCTCTATGCCAACACAGT |  |
